# Impact of early parenteral nutrition on muscle and adipose tissue compartments during critical illness

**DOI:** 10.1186/cc12191

**Published:** 2013-03-19

**Authors:** L Langouche, MP Casaer, W Coudyzer, D Vanbeckevoort, B De Dobbelaer, FG Güiza, PJ Wouters, D Mesotten, G Van den Berghe

**Affiliations:** 1 KU Leuven, Belgium; 2 University Hospitals Leuven - KU Leuven, Belgium

## Introduction

The goal of enhanced nutrition in critically ill patients is to improve outcome by reducing lean tissue wasting. However, such effect has not been proven. This study aimed to assess the effect of early administration of parenteral nutrition (PN) on muscle volume and composition by repeated quantitative computer tomography (qCT).

## Methods

We performed a preplanned substudy of a randomized controlled trial (EPaNIC) that compared early initiation of PN when enteral nutrition was insufficient (early PN) with tolerating a pronounced nutritional deficit for 1 week in the ICU (late PN) [1]. Late PN prevented infections and accelerated recovery. We studied 15 EPaNIC study neurosurgical patients requiring prescheduled repeated follow-up CT scans and six healthy volunteers matched for age, gender and BMI. Repeated abdominal and femoral qCT images were obtained in a standardized manner on median ICU day 2 (IQR 2 to 3) and day 9 (8 to 10). Intramuscular, subcutaneous and visceral fat compartments were delineated manually. Muscle and adipose tissue volume and composition were quantified using standard Hounsfield Unit ranges.

## Results

Critical illness evoked substantial loss of femoral muscle volume in 1 week, irrespective of the nutritional regimen. Early PN reduced the quality of the muscle tissue, as reflected by the attenuation, revealing increased intramuscular water/lipid content. Early PN also increased the volume of adipose tissue islets within the femoral muscle compartment. These changes in skeletal muscle integrity correlated with caloric intake. In the abdominal muscle compartments, changes were similar, albeit smaller. Femoral and abdominal subcutaneous adipose tissue compartments were unaffected by disease and nutritional strategy.

## Conclusion

Early PN did not prevent the pronounced wasting of skeletal muscle observed over the first week of critical illness. Moreover, early PN increased the amount of adipose tissue within the muscle compartments.
